# Supplementary material for: Multi-View Radiomics Feature Fusion Reveals Distinct Immuno-Oncological Characteristics and Clinical Prognoses in Hepatocellular Carcinoma
Source: Cancers (Basel). 2023 Apr 17;15(8):2338. doi: 10.3390/cancers15082338 (PMC10137067; doi:10.3390/cancers15082338)
Supplement: Supplementary file 1 [file cancers-15-02338-s001.zip › Supplymentary Figures.pdf]

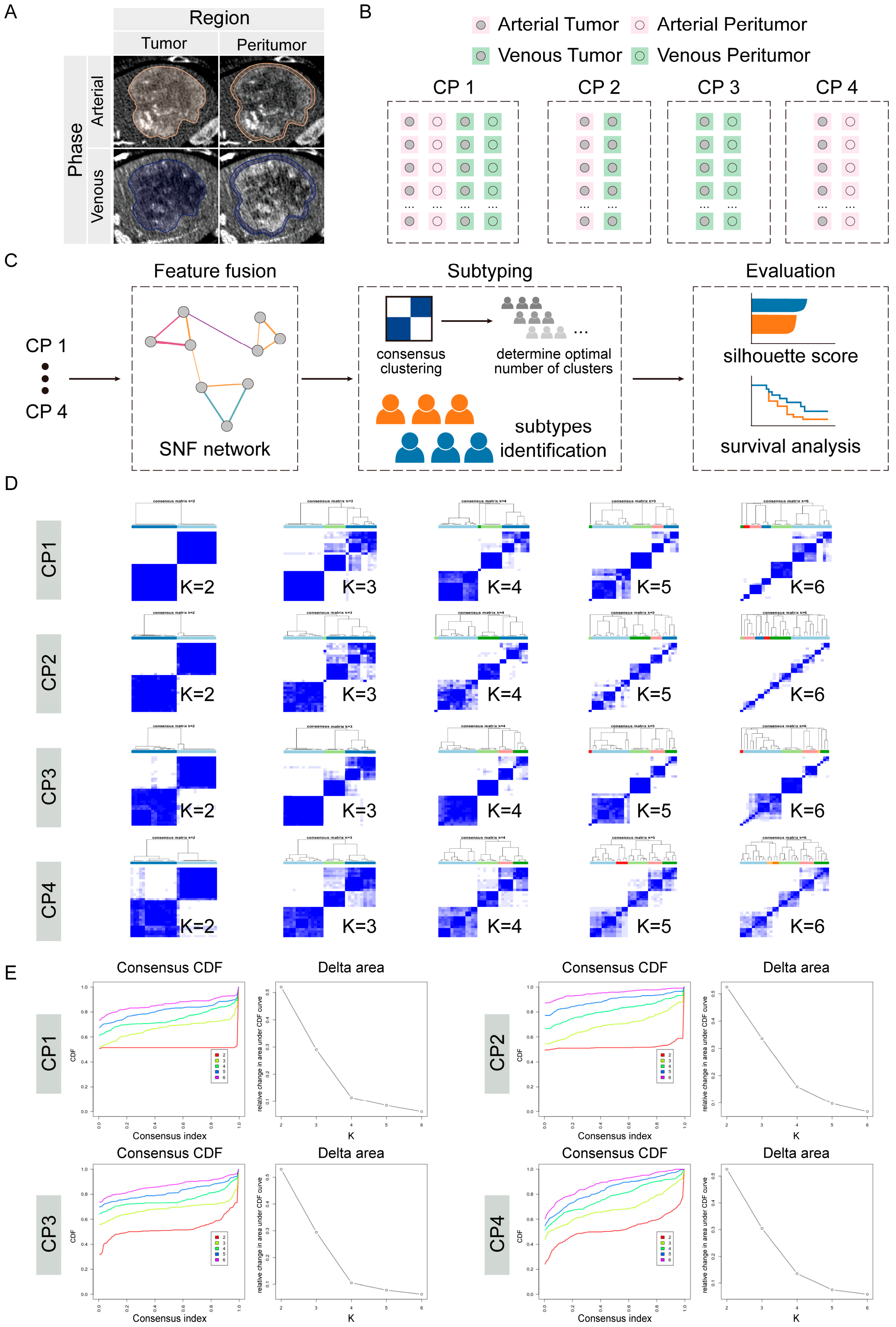

Figure S1. Evaluating the subtyping effects of multi-view radiomics features fusion. (A) Represented multi-view VOI delineation in a patient, including tumoral and peritumoral regions in the arterial phase and venous phase, respectively. The regions in tan represent the tumor and peritumoral VOIs in the arterial phase, and the regions in lightsteelblue represent the tumor and peritumoral VOIs in the venous phase. (B) Four CPs are constructed for the

evaluation of the FIFS model. (C) Workflow for evaluating the FIFS model of fused multi-view radiomics features. The eigenvalue matrices of different CPs are put into the SNF network, followed by consensus clustering to determine the optimal number of subtypes. Additionally, the silhouette scores are calculated to measure the homogeneity of the subtypes, and the Log-rank tests are performed to assess the prognostic outcome of the subtypes. This unified evaluation process is performed in all four CPs. (D) Consensus matrices from each CP show differences in clustering stability when grouped from k=2 to k=6 clusters. (E) Delta plots of the relative change in the area under the CDF curve between clustering numbers in four CPs. CPs, Combine Patterns.

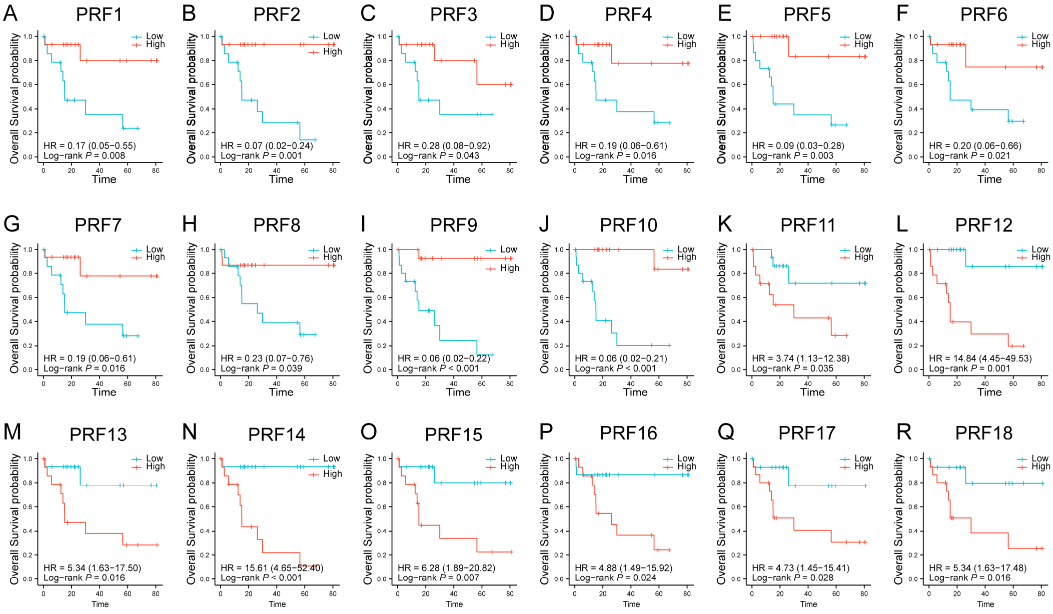

Figure S2. Overall survival analysis of 30 HCC patients according to the median divided eigenvalues for PRFs. (A-R) Kaplan–Meier plots show the prognosis association of PRF1(A)–18(R).

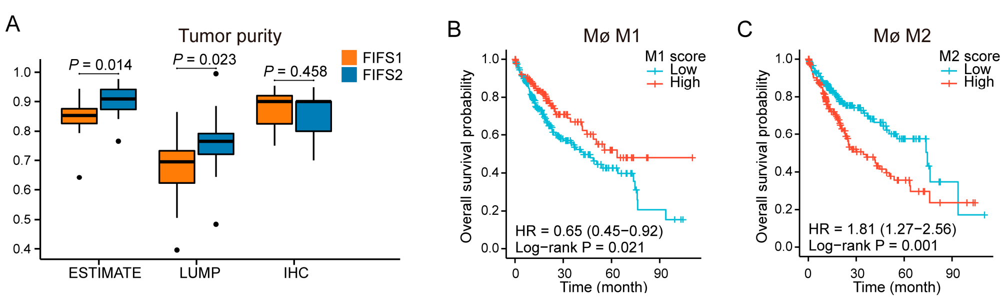

Figure S3. The TIME comparison between radiomics subtypes. (A) The distribution of tumor purity of patients in FIFS1 and FIFS2 is estimated using the ESTIMATE algorithm, the LUMP algorithm, and immunohistochemistry

(IHC) qualitative estimation, respectively. (B-C) The prognostic effects of M1 macrophages (B) and the progression effects of M2 macrophages (C) are shown by the survival analysis. All variables are dichotomized according to the medians. Mø, macrophage.

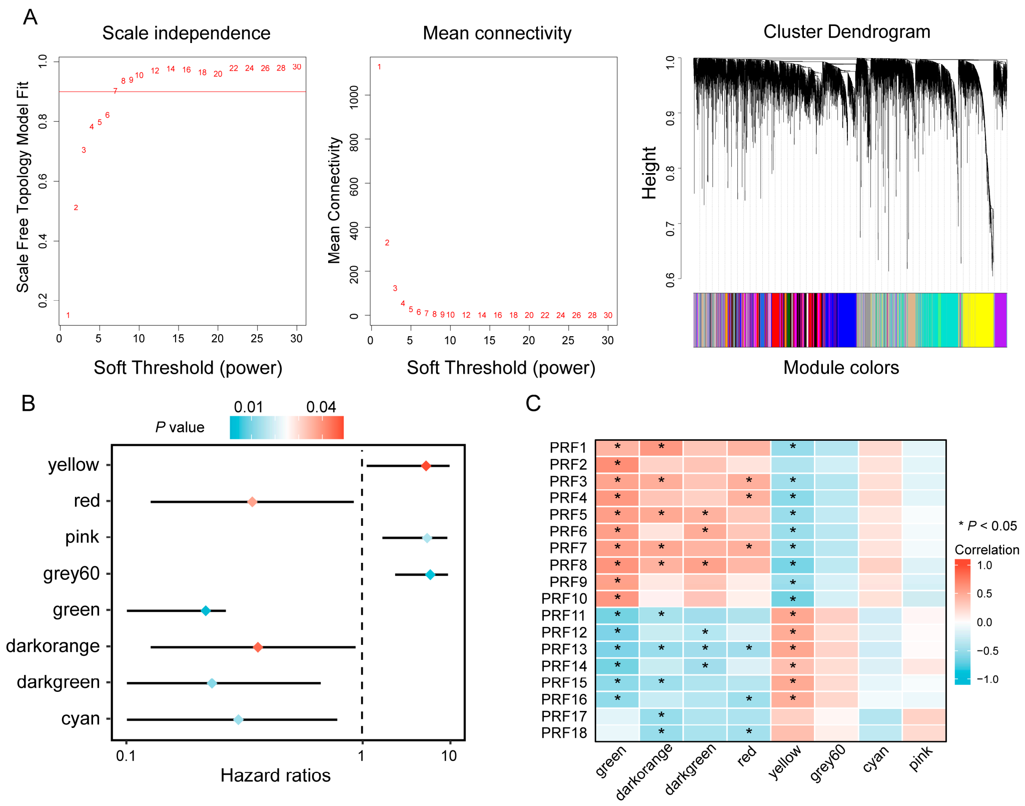

Figure S4. Recognition of subtype-specific co-expression modules. (A) Determination of soft threshold of the adjacency function in the WGCNA algorithm (left and middle), and hierarchical clustering tree of identified co-expression modules (right). (B) A forest plot shows the result of the cox regression analysis of eight subtype-related modules. (C) Correlations between PRFs and eight prognostic modules are displayed as a heatmap.

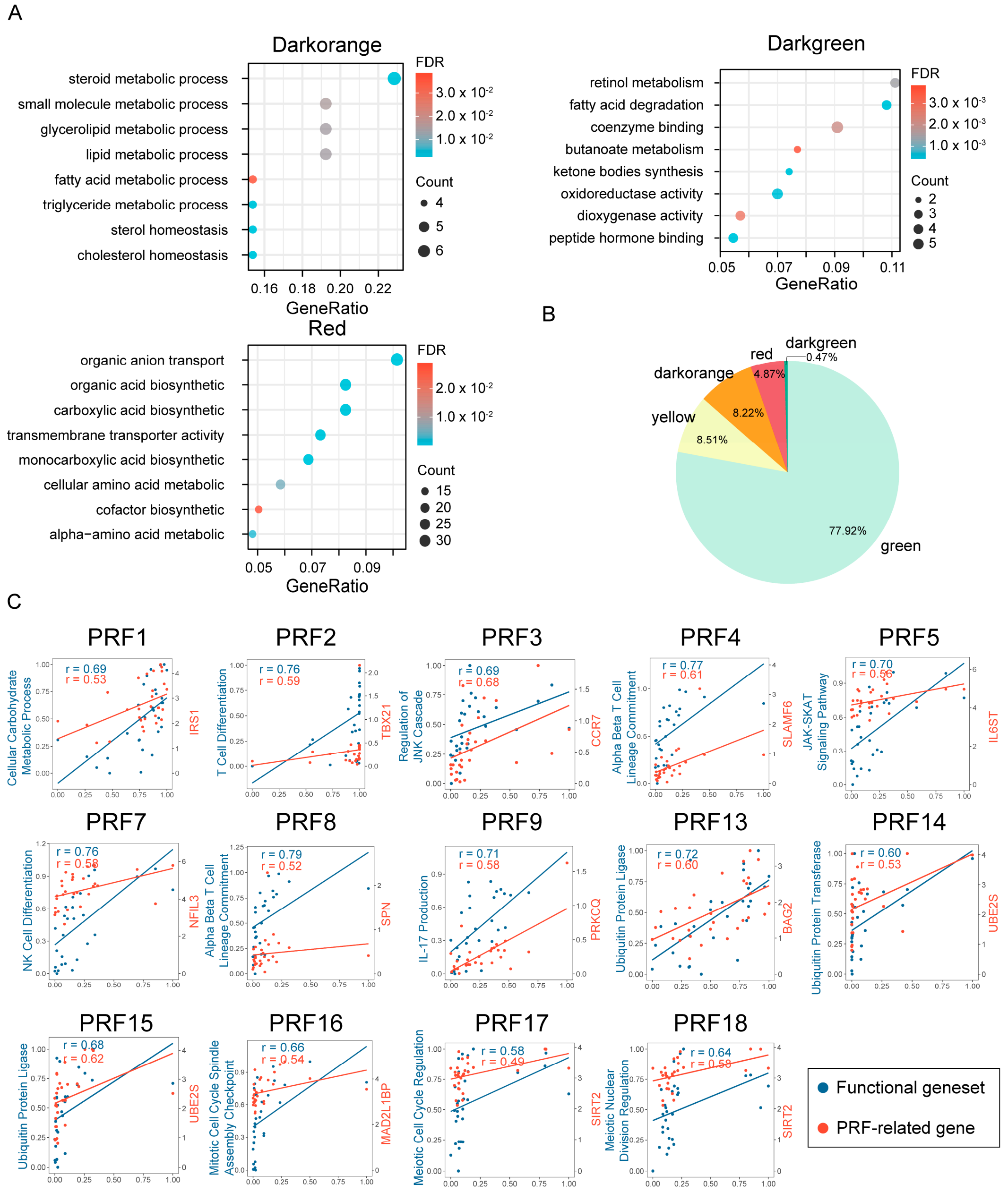

Figure S5. The linkages between biological functions and radiomics features. (A) Enrichment results for the genes of PRF-related modules. (B) A pie chart shows the percentage of pathway-feature pairs in the PRF-related modules. (C) Correlation analysis between the PRFs and biological functions. The points in blue color indicate the relationships between radiomics features and functional genesets, and the points in red color indicate the relationships between radiomics features and the PRF-related genes.

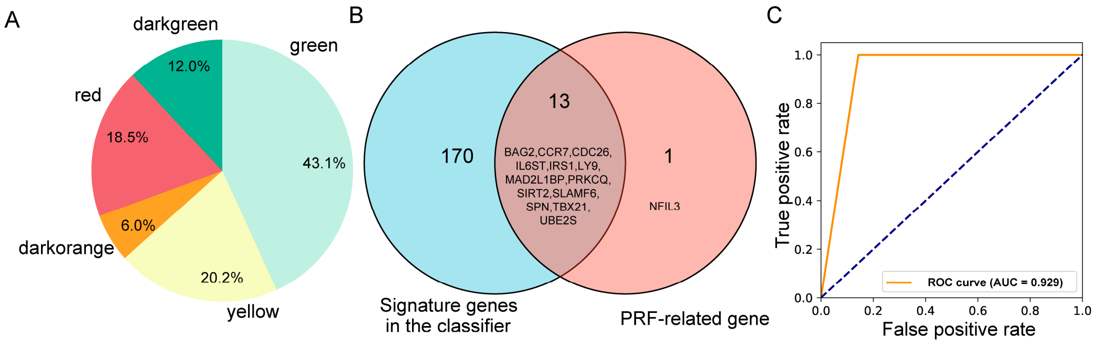

Figure S6. Radiogenomics association validation based on the expression-based classifier. (A) The percentage of the signature genes distributed in the PRF-related modules. (B) Venn diagram shows the overlap genes between the PRF-related genes and the signature genes identified in the nearest shrunken centroid classifier. (C) Receiver operating characteristic (ROC) curve shows the radiomics feature-based random forest model performance in leave-one-out cross-validation.
